# Causal association between immune cells and lung cancer risk: a two-sample bidirectional Mendelian randomization analysis

**DOI:** 10.3389/fimmu.2024.1433299

**Published:** 2024-06-19

**Authors:** Shengshan Xu, Huiying Fang, Tao Shen, Yufu Zhou, Dongxi Zhang, Yongwen Ke, Zhuowen Chen, Zhuming Lu

**Affiliations:** ^1^ Department of Thoracic Surgery, Jiangmen Central Hospital, Jiangmen, Guangdong, China; ^2^ Department of Breast Cancer Center, Chongqing University Cancer Hospital, Chongqing, China; ^3^ Chongqing Key Laboratory for Intelligent Oncology in Breast Cancer (iCQBC), Chongqing University Cancer Hospital, Chongqing, China

**Keywords:** Mendelian randomization, lung cancer, immune cells, causal relationship, genome-wide association studies

## Abstract

**Background:**

Previous studies have highlighted the crucial role of immune cells in lung cancer development; however, the direct link between immunophenotypes and lung cancer remains underexplored.

**Methods:**

We applied two-sample Mendelian randomization (MR) analysis, using genetic variants as instruments to determine the causal influence of exposures on outcomes. This method, unlike traditional randomized controlled trials (RCTs), leverages genetic variants inherited randomly at conception, thus reducing confounding and preventing reverse causation. Our analysis involved three genome-wide association studies to assess the causal impact of 731 immune cell signatures on lung cancer using genetic instrumental variables (IVs). We initially used the standard inverse variance weighted (IVW) method and further validated our findings with three supplementary MR techniques (MR–Egger, weighted median, and MR-PRESSO) to ensure robustness. We also conducted MR–Egger intercept and Cochran’s Q tests to assess heterogeneity and pleiotropy. Additionally, reverse MR analysis was performed to explore potential causality between lung cancer subtypes and identified immunophenotypes, using R software for all statistical calculations.

**Results:**

Our MR analysis identified 106 immune signatures significantly associated with lung cancer. Notably, we found five suggestive associations across all sensitivity tests (*P*<0.05): CD25 on IgD- CD24- cells in small cell lung carcinoma (OR_IVW_ =0.885; 95% CI: 0.798–0.983; *P*_IVW_ =0.022); CD27 on IgD+ CD24+ cells in lung squamous cell carcinoma (OR_IVW_ =1.054; 95% CI: 1.010–1.100; *P*_IVW_ =0.015); CCR2 on monocyte cells in lung squamous cell carcinoma (OR_IVW_ =0.941; 95% CI: 0.898–0.987; *P*_IVW_ =0.012); CD123 on CD62L+ plasmacytoid dendritic cells (OR_IVW_ =0.958; 95% CI: 0.924–0.992; *P*_IVW_ =0.017) as well as on plasmacytoid dendritic cells (OR_IVW_ =0.958; 95% CI: 0.924–0.992; *P*_IVW_ =0.017) in lung squamous cell carcinoma.

**Conclusion:**

This study establishes a significant genomic link between immune cells and lung cancer, providing a robust basis for future clinical research aimed at lung cancer management.

## 1 Introduction

Lung cancer remains one of the most prevalent malignancies globally, ranking second in incidence and leading in cancer-related deaths (1–3). Due to its significant incidence and mortality, lung cancer represents a critical public health challenge, emphasizing the need for effective preventive strategies (4, 5). Identifying potential causal relationships between risk factors and lung cancer is essential for developing these strategies.

Recent advancements in tumor immunology have underscored the importance of understanding the role of immune cells within the lung cancer microenvironment. This understanding is crucial for advancing immunotherapy drug development. The immune system plays a complex role in tumorigenesis; it can suppress tumor growth by eliminating cancer cells, yet it can also promote tumor progression by providing growth and survival factors. For instance, the presence of CD3+ tumor-infiltrating lymphocytes is associated with improved overall survival (OS) in non-small cell lung cancer (NSCLC) (6) and hepatocellular carcinoma (7). Elevated levels of FoxP3+ Tregs are linked to poorer outcomes in several cancers, including melanoma and breast cancer. Conversely, an improvement in OS has been reported in colorectal and head and neck cancers, with variable results in lung cancer regarding disease-free survival (DFS) (8). B-cell infiltration has shown mixed outcomes across different cancers, enhancing survival in breast cancer (9) but presenting inconsistent results in melanoma, hepatocellular carcinoma, ovarian and head and neck cancers (10). Despite significant progress in immune cell research, the links between immunophenotypes and lung cancer remain inconsistent, often limited by small sample sizes, study design flaws, and unaddressed confounders (11–13). The introduction of genome-wide association studies (GWAS) has been transformative, providing new pathways to investigate cancer etiology (14, 15).

In this context, Mendelian randomization (MR), which uses genetic variants as instrumental variables (IVs) to establish causal relationships between exposures and outcomes, offers a powerful epidemiological tool (16). MR is advantageous because it uses genotypes that are fixed at conception, thus reducing bias from confounding factors and reverse causation (17, 18). This study employs a two-sample MR approach, using single nucleotide polymorphisms (SNPs) to evaluate the causal impact of immune cells on lung adenocarcinoma (LUAD), lung squamous cell carcinoma (LUSC), and small cell lung carcinoma (SCLC).

## 2 Materials and methods

### 2.1 Study design

**Figure 1:**
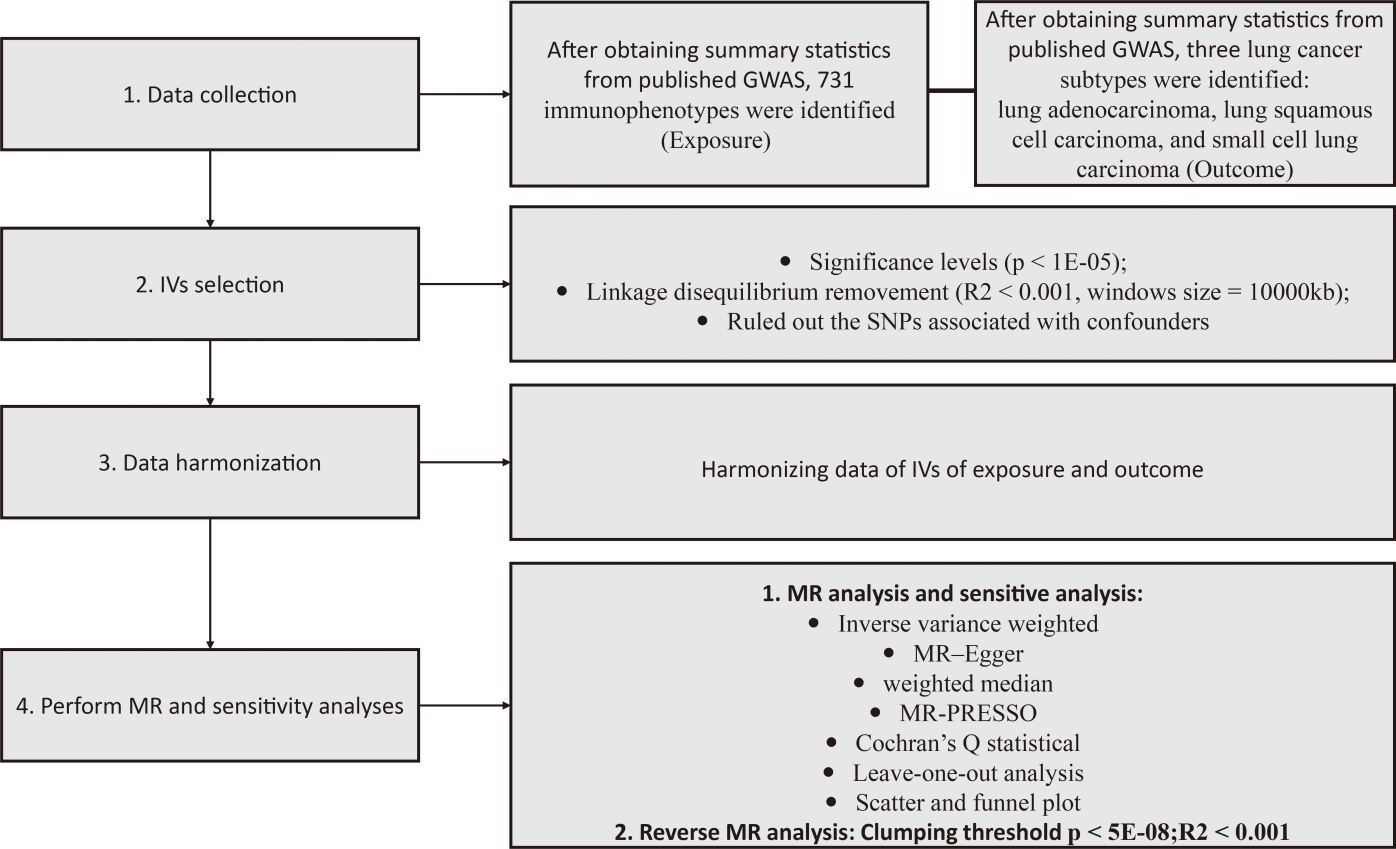
Illustrative schematic of the study methodology. GWAS, genome-wide association study; MR, Mendelian randomization; MR-PRESSO, MR pleiotropy residual sum and outlier test; IV, instrumental variables.

Our two-sample MR study design is depicted in **Figure 1**. The validity of our MR analysis was ensured by meeting three essential criteria: the first criterion confirmed a significant link between the IVs and immunophenotypes. Second, the IVs were free from any relationships with confounding elements. Finally, outside of exposure elements, there was no impact of the IVs on outcomes through other pathways (19).

### 2.2 Genome-wide association study data sources for lung cancer

We obtained GWAS summary data for lung adenocarcinoma (LUAD), lung squamous cell carcinoma (LUSC), and small cell lung carcinoma (SCLC) from J. D. McKay et al. (20) via the IEU-OpenGWAS platform. The study involved 21,363 lung cancer patients, namely, 11,273 LUAD, 7,426 LUSC and 2,664 SCLC patients and 55,483, 55,627, and 21,444 controls (**Supplementary Table 1**). In the quality assurance stage, SNPs exhibiting suboptimal imputation (R2 < 0.3 or Info < 0.4) or a minor allele frequency greater than 0.01 were excluded. Approximately 8 million SNPs were retained for the GWAS.

### 2.3 Sources of immunity-wide GWAS data

We sourced comprehensive GWAS data for 731 immunophenotypes from the largest study to date, involving 3,757 Europeans (21). Approximately 22 million SNPs, adjusted for sex and age (including age squared), were genotyped with high-density arrays and imputed employing a reference panel based on Sardinian sequences (22).

### 2.4 Selection criteria for IVs for 731 immunophenotypes

To identify sufficient SNPs (number >3) for both exposure and outcome analyses, we selected SNPs with genome-wide suggestive significance (*P*<1×10^-5^). This method is frequently utilized in MR research as it encompasses a wider array of variations, particularly when there are limited genome-wide significant SNPs available for analysis (23). Independent SNPs were identified using a clumping process with stringent criteria (r2 < 0.001, window size 10,000 kb) using the European 1000 Genomes reference panel (17). Following steps evaluated the robustness of these IVs in predicting causal effects using the F-test (24). The formula used in the design is outlined in **Supplementary Table 2**. An F-statistic greater than 10 typically signifies strong IVs, and any immunophenotypes with an F-statistic below 10 were discarded (25). The PhenoScannerV2 database (http://www.phenoscanner.medschl.cam.ac.uk/) was employed to identify and remove SNPs directly linked to cancer and other recognized confounders in cancer progression, such as smoking (26, 27) and alcohol consumption (28). In the reverse MR analysis, the threshold for statistical significance was established at *P* < 5 × 10^–8^, using a clumping parameter analogous to that used in the forward-direction analysis.

### 2.5 MR statistical analysis

In this MR study, we investigated the causal associations between immune cell profiles and different subtypes of lung cancer (LUAD, LUSC, SCLC) using the standard inverse variance weighted (IVW) approach. We also applied MR-Egger and weighted median methods as supplementary analyses to IVW, especially in scenarios where a significant fraction of variants (up to 50% or less) might originate from potentially invalid IVs (29, 30). Results were presented as odds ratios (ORs) with 95% confidence intervals (CIs). To identify any potential horizontal pleiotropy or outliers among the SNPs, we implemented the MR-Egger intercept test (29) and the Mendelian Randomization Pleiotropy Residual Sum and Outlier (MR-PRESSO) test (31). The reliability of our MR results was verified using the Cochran Q statistic to evaluate SNP heterogeneity (32). A sensitivity analysis called “leave-one-out” was conducted, where SNPs were sequentially removed. This analysis was complemented by applying the IVW-random method to the remaining set of SNPs to determine the influence of outlying variants on the findings (33). For thorough analysis of heterogeneity, we generated forest and scatter plots. A meta-analysis was then undertaken to elucidate the causal connections between the identified immunophenotypes and lung cancer subtypes by synthesizing MR data from two distinct cohorts (34). In instances of significant heterogeneity or pleiotropy, adjustments were made to the ORs and CIs for the meta-analysis. Based on the degree of heterogeneity observed, the choice was between a fixed-effects model (I2 ≤ 50%) and a random-effects model (I2 > 50%). The conclusions from the meta-analysis were considered the definitive causal relationships (35). To address concerns of multiple testing, a Bonferroni-corrected significance threshold of 6.84 × 10^-5^ (0.05/731 for the 731 immunophenotypes evaluated) was employed. P values between 6.84 × 10–5 and 0.05 were deemed indicative of suggestive causal links between the exposures and outcomes. All analyses were conducted using the “TwoSampleMR” and “MRPRESSO” packages in R (version 4.2.0).

## 3 Results

### 3.1 Selection of IVs

Our two-sample MR analysis identified 106 immunophenotypes with IVs ranging from 4 to 103 SNPs, indicating suggestive associations at P<0.05. The IVs for each phenotype showed high potency, with F-statistics ranging from 19.546 upwards, confirming their reliability for MR studies.

### 3.2 Causal effects of immunophenotypes on 3 lung cancer subtypes

**Figure 2:**
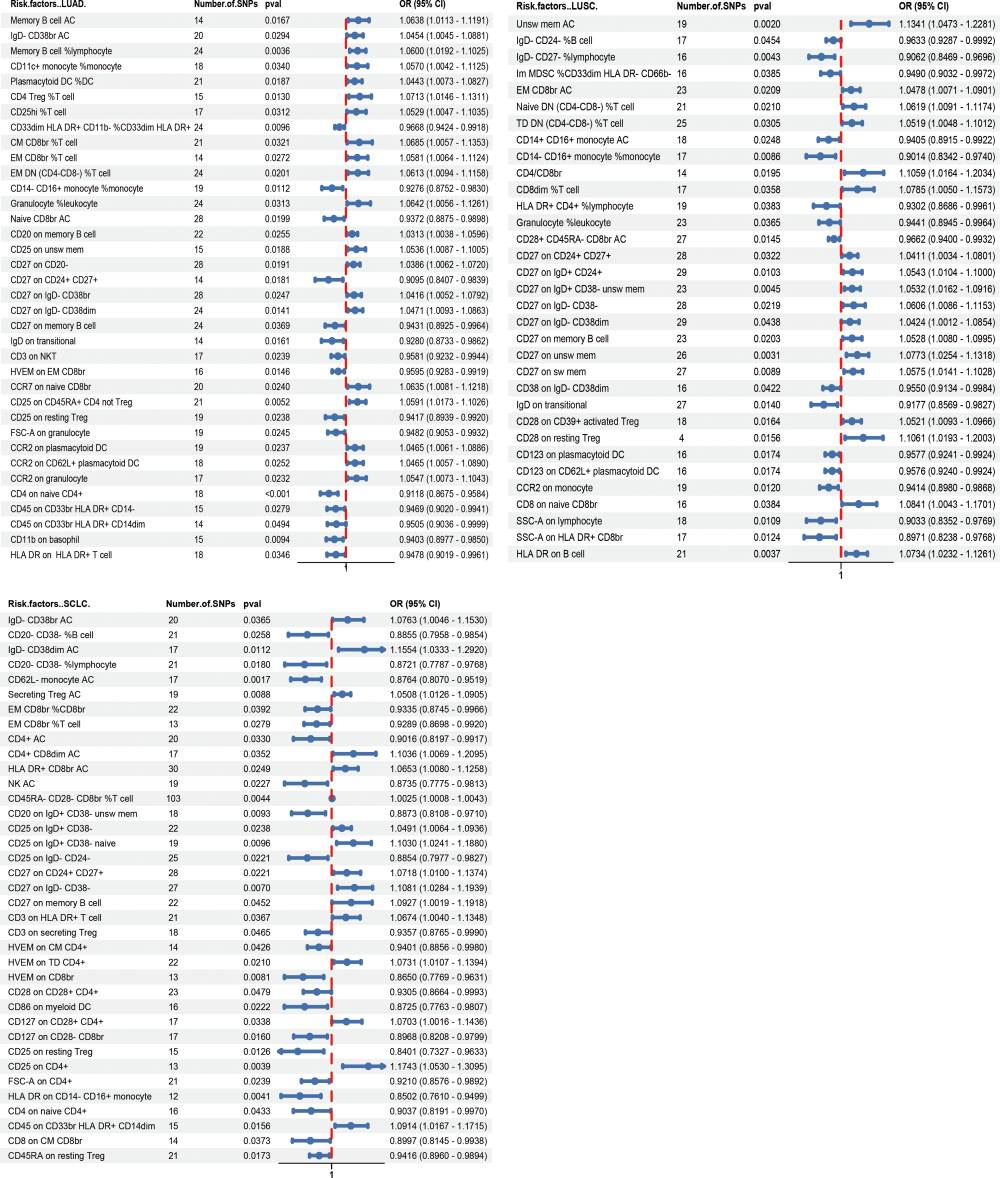
Forest plot depicting the Mendelian randomization analyses for associations between various immunophenotypes and lung cancer subtypes.

Using the IVW method, significant associations were found between 36 immunophenotypes and lung adenocarcinoma (LUAD), 33 with lung squamous cell carcinoma (LUSC), and 37 with small cell lung carcinoma (SCLC), as detailed in **Figure 2** and **Supplementary Table 3**. Notably, certain immunophenotypes such as CD4 Treg %T cells in LUAD (OR_IVW_ =1.071; 95% CI: 1.015–1.131; *P*_IVW_ =0.013), Unsw mem AC in LUSC (OR_IVW_ =1.134; 95% CI: 1.047–1.228; *P*_IVW_ =0.002), and CD25 on CD4+ T cells in SCLC (OR_IVW_ =1.174; 95% CI: 1.053–1.310; *P*_IVW_ =0.004) were associated with increased risk, while CD27 on IgD- CD38br cells (OR_IVW_ =0.909; 95% CI: 0.841–0.984; *P*_IVW_ =0.018), SSC-A on HLA DR+ CD8br cells (OR_IVW_ =0.897; 95% CI: 0.824–0.977; *P*_IVW_ =0.012) and CD25 on resting Treg cells (OR_IVW_ =0.840; 95% CI: 0.733–0.963; *P*_IVW_ =0.013) showed protective effects across different subtypes (**Table 1**; **Supplementary Figures 1**-**4**). Additionally, the presence of CD27 on CD24+ CD27+ cells was associated with an increased risk across all three lung cancer subtypes (for LUAD, OR_IVW_ =1.039; 95% CI: 1.006–1.072; *P*_IVW_ =0.019; for LUSC, OR_IVW_ =1.041; 95% CI: 1.003–1.080; *P*_IVW_ =0.032; for SCLC, OR_IVW_ =1.072; 95% CI: 1.010–1.137; *P*_IVW_ =0.022). Similarly, CD27 on memory B cells also showed increased risks for lung cancer subtypes (for LUAD, OR_IVW_ =1.047; 95% CI: 1.009–1.086; *P*_IVW_ =0.014; for LUSC, OR_IVW_ =1.053; 95% CI: 1.008–1.099; *P*_IVW_ =0.020; for SCLC, OR_IVW_ =1.093; 95% CI: 1.002–1.192; *P*_IVW_ =0.045). The results imply a shared biological pathway among these subtypes of lung cancer, influenced by CD27 expression on CD24+ CD27+ cells and memory B cells, as outlined in **Table 2** and **Supplementary Figures 5**-**8**. The genetic variants that clarify the links between these immunophenotypes and lung cancer are detailed in **Supplementary Tables 4**-**15**.

**Table 1:** The most detrimental and protective factors for lung cancer subtypes.

Trait	Exposure	IVW		MR-Egger		Weighted median	
		OR (95% CI)	P-value	OR (95% CI)	P-value	OR (95% CI)	P-value
Lung adenocarcinoma	CD4 Treg %T cells	1.071(1.015-1.131)	0.013	1.044(0.965-1.129)	0.303	1.028(0.960-1.101)	0.427
Lung squamous cell carcinoma	Unsw mem AC	1.134(1.047-1.228)	0.002	1.050(0.862-1.280)	0.634	1.075(0.961-1.203)	0.205
Small cell lung carcinoma	CD25 on CD4+ T cells	1.174(1.053-1.310)	0.004	0.985(0.829-1.170)	0.866	1.195(1.037-1.378)	0.014
Lung adenocarcinoma	CD27 on IgD- CD38br cells	0.909(0.841-0.984)	0.018	0.869(0.608-1.243)	0.457	0.915(0.823-1.019)	0.105
Lung squamous cell carcinoma	SSC-A on HLA DR+ CD8br cells	0.897(0.824-0.977)	0.012	0.973(0.848-1.117)	0.703	0.954(0.860-1.059)	0.379
Small cell lung carcinoma	CD25 on resting Treg cells	0.840(0.733-0.963)	0.013	0.794(0.516-1.223)	0.315	0.772(0.639**-**0.934)	0.008

**Table 2:** Causal effects between CD27 on CD24+ CD27+ cells and CD27 on memory B cells with lung cancer subtypes.

Trait	Exposure	IVW		MR-Egger		Weighted median	
		OR (95% CI)	P-value	OR (95% CI)	P-value	OR (95% CI)	P-value
Lung adenocarcinoma	CD27 on CD24+ CD27+ cells	1.039(1.006**-**1.072)	0.019	1.045(0.992**-**1.101)	0.113	1.035(0.985**-**1.088)	0.175
Lung squamous cell carcinoma	CD27 on CD24+ CD27+ cells	1.041(1.003**-**1.080)	0.032	1.021(0.961**-**1.085)	0.507	1.042(0.987**-**1.099)	0.135
Small cell lung carcinoma	CD27 on CD24+ CD27+ cells	1.072(1.010**-**1.137)	0.022	1.021(0.923**-**1.130)	0.689	1.026(0.941**-**1.118)	0.563
Lung adenocarcinoma	CD27 on memory B cells	1.047(1.009**-**1.086)	0.014	1.037(0.978**-**1.100)	0.235	1.043(0.993**-**1.097)	0.093
Lung squamous cell carcinoma	CD27 on memory B cells	1.053(1.008**-**1.099)	0.02	1.057(0.986**-**1.133)	0.134	1.074(1.012**-**1.140)	0.018
Small cell lung carcinoma	CD27 on memory B cells	1.093(1.002**-**1.192)	0.045	1.089(0.946**-**1.254)	0.248	1.077(0.980**-**1.183)	0.123

### 3.3 Sensitivity and pleiotropy analysis

**Figure 3:**
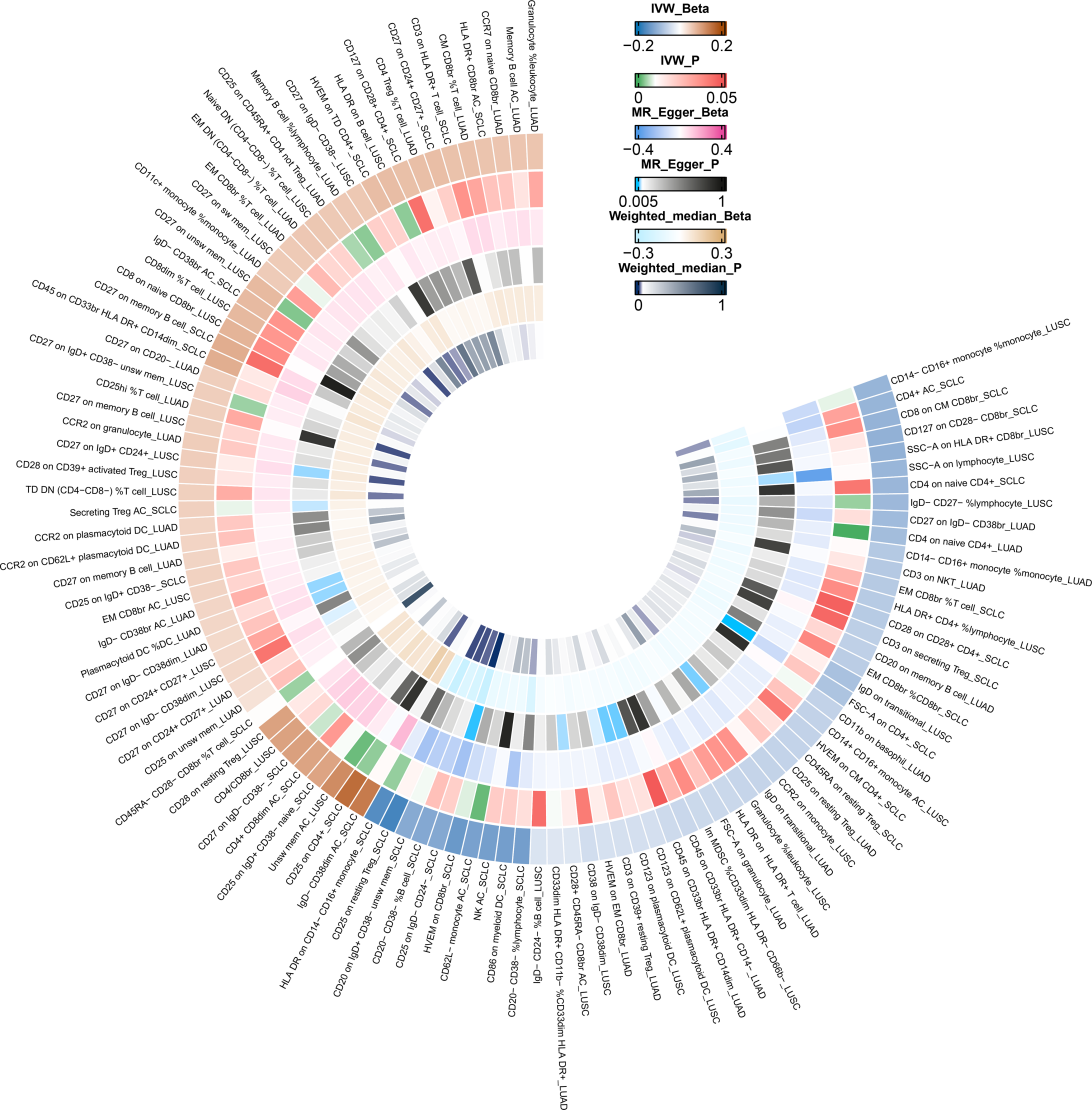
IVW Mendelian randomization estimates, MR–Egger estimates, and weighted-median estimates for the associations between immunophenotypes and lung cancer subtypes. IVW, inverse variance weighted; MR, Mendelian randomization.

Due to potential biases from weak instruments in the IVW approach, we expanded our study to incorporate additional sensitivity and pleiotropy assessments, with detailed findings listed in **Supplementary Table 3**. Noteworthy, pleiotropic effects were observed for SSC-A on HLA DR+ CD8br cells in LUSC (P_MR-PRESSO_ Global =0.039). The combined outcomes from IVW, MR-Egger, and weighted median methods across immunophenotypes with suggestive links are displayed in **Figure 3**. Furthermore, we discerned five immunophenotypes with suggestive links that passed all sensitivity analyses (*P*<0.05) (**Table 3**; **Supplementary Figures 9**-**12**): CD25 on IgD- CD24- cells in SCLC (OR_IVW_ =0.885; 95% CI: 0.798–0.983; *P*_IVW_ =0.022), CD27 on IgD+ CD24+ cells in LUSC (OR_IVW_ =1.054; 95% CI: 1.010–1.100; *P*_IVW_ =0.015), CCR2 on monocyte cells in LUSC (OR_IVW_ =0.941; 95% CI: 0.898–0.987; *P*_IVW_ =0.012), CD123 on CD62L+ plasmacytoid dendritic cells (DCs) of LUSC (OR_IVW_ =0.958; 95% CI: 0.924–0.992; *P*_IVW_ =0.017), and CD123 on plasmacytoid DCs of LUSC (OR_IVW_ =0.958; 95% CI: 0.924–0.992; *P*_IVW_ =0.017). Additional validation through MR analysis utilized GWAS data for SCLC (ieu-a-988: 2,791 patients and 20,580 controls) and LUSC (ieu-a-989: 7,704 patients and 54,763 controls), with results detailed in **Supplementary Tables 16**, **17**. Genetic variants clarifying the associations between these five immunophenotypes and lung cancer are summarized in **Supplementary Tables 18**-**22**. In reverse MR analyses, a suggestive link was observed for LUSC risk and CCR2 on monocyte cells (OR_IVW_ =0.888; 95% CI: 0.790–0.999; *P*_IVW_ =0.048). Lung cancer subtypes with at least two robust MR findings were included in the meta-analysis, whose results are compiled in **Supplementary Table 23**. Four immunophenotypes demonstrated a suggestive correlation with LUSC risk: CD27 on IgD+ CD24+ cells (OR = 1.0567; 95% CI: 1.0263 to 1.0880; *P* = 0.0002), CCR2 on monocyte cells (OR = 0.9483; 95% CI: 0.9238 to 0.9735; *P* < 0.0001), CD123 on CD62L+ plasmacytoid DCs (OR = 0.9629; 95% CI: 0.9414 to 0.9850; *P* = 0.0011), and CD123 on plasmacytoid DCs (OR = 0.9630; 95% CI: 0.9414 to 0.9850; *P* = 0.0011). Additionally, CD25 on IgD- CD24- cells was linked to a decreased risk of SCLC (OR = 0.8701; 95% CI: 0.8175 to 0.9260; *P* < 0.0001). The findings indicate the reliability of the causal relationship between the identified immune phenotype and subtypes of lung cancer.

**Table 3:** Statistically significant association between five potential immune cell signatures and lung cancer.

Trait	Exposure	IVW		MR-Egger		Weighted median	
		OR (95% CI)	P-value	OR (95% CI)	P-value	OR (95% CI)	P-value
Small cell lung carcinoma	CD25 on IgD- CD24- cells	0.885(0.798**-**0.983)	0.022	0.790(0.676**-**0.923)	0.007	0.772(0.672**-**0.887)	0
Lung squamous cell carcinoma	CD27 on IgD+ CD24+ cells	1.054(1.010**-**1.100)	0.015	1.081(1.015**-**1.152)	0.023	1.079(1.018**-**1.144)	0.011
Lung squamous cell carcinoma	CCR2 on monocyte cells	0.941(0.898**-**0.987)	0.012	0.919(0.864**-**0.978)	0.017	0.928(0.868**-**0.992)	0.029
Lung squamous cell carcinoma	CD123 on CD62L+ plasmacytoid dendritic cells	0.958(0.924**-**0.992)	0.017	0.938(0.896**-**0.981)	0.014	0.950(0.904**-**0.999)	0.047
Lung squamous cell carcinoma	CD123 on plasmacytoid dendritic cells	0.958(0.924**-**0.992)	0.017	0.938(0.896**-**0.981)	0.014	0.950(0.903**-**1.000)	0.049

## 4 Discussion

This MR study marks a significant advance in understanding the causal effects of immune cell signatures on lung cancer, focusing on three specific subtypes. Leveraging a robust two-sample MR framework that incorporates IVW, MR-Egger, and weighted median approaches, our study advances beyond earlier observational research that predominantly concentrated on correlations (36, 37). By utilizing the most comprehensive GWAS datasets currently available for the immunophenotyping of peripheral blood, our research significantly enhances the investigation into the connections between immune cells and disease, expanding the scope further than prior studies (38, 39). Moreover, we utilized meta-analysis to consolidate data from multiple studies, thereby enhancing the robustness of our conclusions. The discovery of 106 immune signatures, particularly five key associations such as CD25 on IgD- CD24- cells in SCLC and CCR2 on monocyte cells in LUSC, enriches our understanding of these cells’ causal involvement in lung cancer.

This research offers insightful hypotheses regarding the mechanistic roles of these immune signatures in lung cancer. The diverse interactions of immune cell subsets within the tumor microenvironment hint at their potential influence on tumor growth, apoptosis, and microenvironment dynamics. The distinct responses observed across lung cancer subtypes emphasize the specificity of immune reactions and suggest potential avenues for therapeutic intervention. Of the 106 immune signatures studied, five showed significant links to lung cancer subtypes, including CD25 on IgD- CD24- cells in SCLC, CD27 on IgD+ CD24+ cells in LUSC, CCR2 on monocyte cells in LUSC, CD123 on CD62L+ plasmacytoid DCs in LUSC, and CD123 on plasmacytoid DCs in LUSC, pointing to their roles in cancer development.

Significantly, our results emphasize the association of CCR2 with monocyte cells in LUSC. CCR2-positive monocytes are attracted to the LUSC tumor microenvironment in response to signals from cancer-associated fibroblasts via CCL2, contributing to an immunosuppressive environment (40, 41). Additionally, these monocytes, once present in inflamed lung areas, tend to reduce local CCL2 levels (42). Concurrent studies, like that by Lei Li and colleagues, have shown high CCL2 levels in the tumor microenvironment as predictors of survival in lung cancer patients (43). There is also evidence that CD24 facilitates interactions among B cells, with CD24-deficient mice displaying B-cell anomalies (44). High CD24 levels have been identified as adverse prognostic factors for progression-free and cancer-specific survival in NSCLC patients (45, 46). Moreover, this research highlights the essential role of DCs in LUSC, where tumor-infiltrating mature DCs correlate with better NSCLC prognosis (47, 48).

Despite its strengths, this study has limitations. Firstly, the cohort comprised mainly European individuals, which might limit the generalizability of the findings to more diverse populations. Second, the selection criteria for IVs were relatively permissive, establishing a significance level at *P* < 1 × 10^−5^, potentially leading to the incorporation of false-positive variants, potentially introducing bias into the results. Nevertheless, the F-statistics for all IVs exceeded 10, mitigating the concern for weak instrument bias. Third, despite our thorough examination for possible secondary phenotypes of IVs and the ability to conduct multiple sensitivity analyses, the potential for pleiotropy cannot be entirely dismissed. Fourthly, no immunophenotypes showed a statistically significant association with lung cancer risk after Bonferroni correction.

With further validation in larger populations and additional SNP analysis, identifying these immune signatures as biomarkers could enhance risk prediction, early detection, and prevention strategies in clinical settings. These advances may pave the way for more personalized cancer treatments. Additionally, the identified immune cells serve as promising targets for experimental investigation to determine their impact on lung cancer and the development of innovative immunotherapies. Specifically, focusing on the pathways that regulate these immune cells might facilitate the development of new immunotherapies for lung cancer. As immunotherapy increasingly becomes a cornerstone of cancer therapy, our results could provide significant contributions to the domain.

In summary, our research offers critical insights into the links between immune signatures and lung cancer, potentially leading to new therapeutic strategies. Continued investigation is essential to fully decipher these interactions and their implications for treating and preventing lung cancer.

## 5 Conclusion

In conclusion, this investigation marks the first comprehensive MR study to explore the causal links between immunophenotypes and specific lung cancer subtypes using genome-wide data, providing initial insights into how immune cell signatures might affect lung cancer risk. Utilizing the IVW method and various sensitivity analyses, we identified strong associations between specific immune signatures such as CD25 on IgD- CD24- cells, CD27 on IgD+ CD24+ cells, CCR2 on monocyte cells, and CD123 on both CD62L+ and plasmacytoid dendritic cells with the development of lung cancer. Our results indicate that these immune cell signatures hold potential as valuable biomarkers for the early detection and prevention of lung cancer in clinical settings. These insights open avenues for further studies aimed at understanding the mechanisms through which these immune cells influence lung cancer and developing targeted therapies. While our study has successfully linked numerous immune cell signatures with the incidence of lung cancer, additional research is required to fully understand their roles in the pathogenesis of lung tumors.

## Data availability statement

The original contributions presented in the study are included in the article/**Supplementary Material**. Further inquiries can be directed to the corresponding authors.

## Ethics statement

SX, HF, TS, YZ, DZ, YK, ZC, and ZL assure that, for the manuscript “Causal association between immune cells and lung cancer risk: a two-sample bidirectional Mendelian randomization analysis”, the following is fulfilled: 1) This material is the authors’ original work, which has not been previously published elsewhere. 2) The paper is not currently being considered for publication elsewhere. 3) The paper reflects the authors’ research and analysis truthfully and completely. 4) The paper properly credits the meaningful contributions of co-authors. 5) All the authors have been personally and actively involved in substantial work leading.

## Author contributions

SX: Conceptualization, Data curation, Funding acquisition, Investigation, Methodology, Software, Writing – original draft, Writing – review & editing. HF: Conceptualization, Funding acquisition, Methodology, Writing – original draft, Writing – review & editing. TS: Formal analysis, Investigation, Writing – review & editing. YZ: Validation, Writing – review & editing. DZ: Writing – review & editing. YK: Writing – review & editing. ZC: Writing – review & editing. ZL: Conceptualization, Funding acquisition, Investigation, Methodology, Supervision, Validation, Writing – original draft, Writing – review & editing.

## Supplementary material

Supplementary Figure 1 Scatter plots illustrating genetic associations of six distinct immunophenotypes with lung cancer risk across different subtypes: **(A)** CD4 Treg %T cells in LUAD, **(B)** Unsw mem AC in LUSC, **(C)** CD25 on CD4+ T cells in SCLC, **(D)** CD27 on IgD- CD38br cells in LUAD, **(E)** SSC-A on HLA DR+ CD8br cells in LUSC, **(F)** CD25 on resting Treg cells in SCLC.

Supplementary Figure 2 Forest plots for six immunophenotypes in lung cancer. **(A)** CD4 Treg %T cells in LUAD, **(B)** Unsw mem AC in LUSC, **(C)** CD25 on CD4+ T cells in SCLC, **(D)** CD27 on IgD- CD38br cells in LUAD, **(E)** SSC-A on HLA DR+ CD8br cells in LUSC, **(F)** CD25 on resting Treg cells in SCLC.

Supplementary Figure 3 Leave-one-out plots for six immunophenotypes in lung cancer. **(A)** CD4 Treg %T cells in LUAD, **(B)** Unsw mem AC in LUSC, **(C)** CD25 on CD4+ T cells in SCLC, **(D)** CD27 on IgD- CD38br cells in LUAD, **(E)** SSC-A on HLA DR+ CD8br cells in LUSC, **(F)** CD25 on resting Treg cells in SCLC.

Supplementary Figure 4 Funnel plots for six immunophenotypes in lung cancer. **(A)** CD4 Treg %T cells in LUAD, **(B)** Unsw mem AC in LUSC, **(C)** CD25 on CD4+ T cells in SCLC, **(D)** CD27 on IgD- CD38br cells in LUAD, **(E)** SSC-A on HLA DR+ CD8br cells in LUSC, **(F)** CD25 on resting Treg cells in SCLC.

Supplementary Figure 5 Scatter plots depicting the genetic correlations between two immune markers and the risk of lung cancer among different subtypes. **(A)** CD27 on CD24+ CD27+ cells in LUAD, **(B)** CD27 on CD24+ CD27+ cells in LUSC, **(C)** CD27 on CD24+ CD27+ cells in SCLC, **(D)** CD27 on memory B cells in LUAD, **(E)** CD27 on memory B cells in LUSC, and **(F)** CD27 on memory B cells in SCLC.

Supplementary Figure 6 Forest plots for assessing the association of two immune phenotypes with lung cancer risk. **(A)** CD27 on CD24+ CD27+ cells in LUAD, **(B)** CD27 on CD24+ CD27+ cells in LUSC, **(C)** CD27 on CD24+ CD27+ cells in SCLC, **(D)** CD27 on memory B cells in LUAD, **(E)** CD27 on memory B cells in LUSC, and **(F)** CD27 on memory B cells in SCLC.

Supplementary Figure 7 Leave-one-out sensitivity plots for two immunophenotypes across lung cancer subtypes. **(A)** CD27 on CD24+ CD27+ cells in LUAD, **(B)** CD27 on CD24+ CD27+ cells in LUSC, **(C)** CD27 on CD24+ CD27+ cells in SCLC, **(D)** CD27 on memory B cells in LUAD, **(E)** CD27 on memory B cells in LUSC, and **(F)** CD27 on memory B cells in SCLC.

Supplementary Figure 8 Funnel plots for two immunophenotypes of lung cancer. **(A)** CD27 on CD24+ CD27+ cells in LUAD, **(B)** CD27 on CD24+ CD27+ cells in LUSC, **(C)** CD27 on CD24+ CD27+ cells in SCLC, **(D)** CD27 on memory B cells in LUAD, **(E)** CD27 on memory B cells in LUSC, and **(F)** CD27 on memory B cells in SCLC.

Supplementary Figure 9 Scatter plots depicting the genetic correlations between four immune markers and the risk of lung cancer among different subtypes. **(A)** CD25 on IgD- CD24- cells in SCLC, **(B)** CD27 on IgD+ CD24+ cells in LUSC, **(C)** CCR2 on monocyte cells in LUSC, and **(D)** CD123 on CD62L+ plasmacytoid dendritic cells in LUSC.

Supplementary Figure 10 Forest plots for assessing the association of five immune phenotypes with lung cancer risk. **(A)** CD25 on IgD- CD24- cells in SCLC, **(B)** CD27 on IgD+ CD24+ cells in LUSC, **(C)** CCR2 on monocyte cells in LUSC, **(D)** CD123 on CD62L+ plasmacytoid dendritic cells in LUSC, and **(E)** CD123 on plasmacytoid dendritic cells in LUSC.

Supplementary Figure 11 Leave-one-out sensitivity plots for five immunophenotypes across lung cancer subtypes. **(A)** CD25 on IgD- CD24- cells in SCLC, **(B)** CD27 on IgD+ CD24+ cells in LUSC, **(C)** CCR2 on monocyte cells in LUSC, **(D)** CD123 on CD62L+ plasmacytoid dendritic cells in LUSC, and **(E)** CD123 on plasmacytoid dendritic cells in LUSC.

Supplementary Figure 12 Funnel plots for five immunophenotypes of lung cancer. **(A)** CD25 on IgD- CD24- cells in SCLC, **(B)** CD27 on IgD+ CD24+ cells in LUSC, **(C)** CCR2 on monocyte cells in LUSC, **(D)** CD123 on CD62L+ plasmacytoid dendritic cells in LUSC, and **(E)** CD123 on plasmacytoid dendritic cells in LUSC.
