# Supplementary figures and images for: Causal association between immune cells and lung cancer risk: a two-sample bidirectional Mendelian randomization analysis
Source: Front Immunol. 2024 Jun 19;15:1433299. doi: 10.3389/fimmu.2024.1433299 (PMC11219561; doi:10.3389/fimmu.2024.1433299)

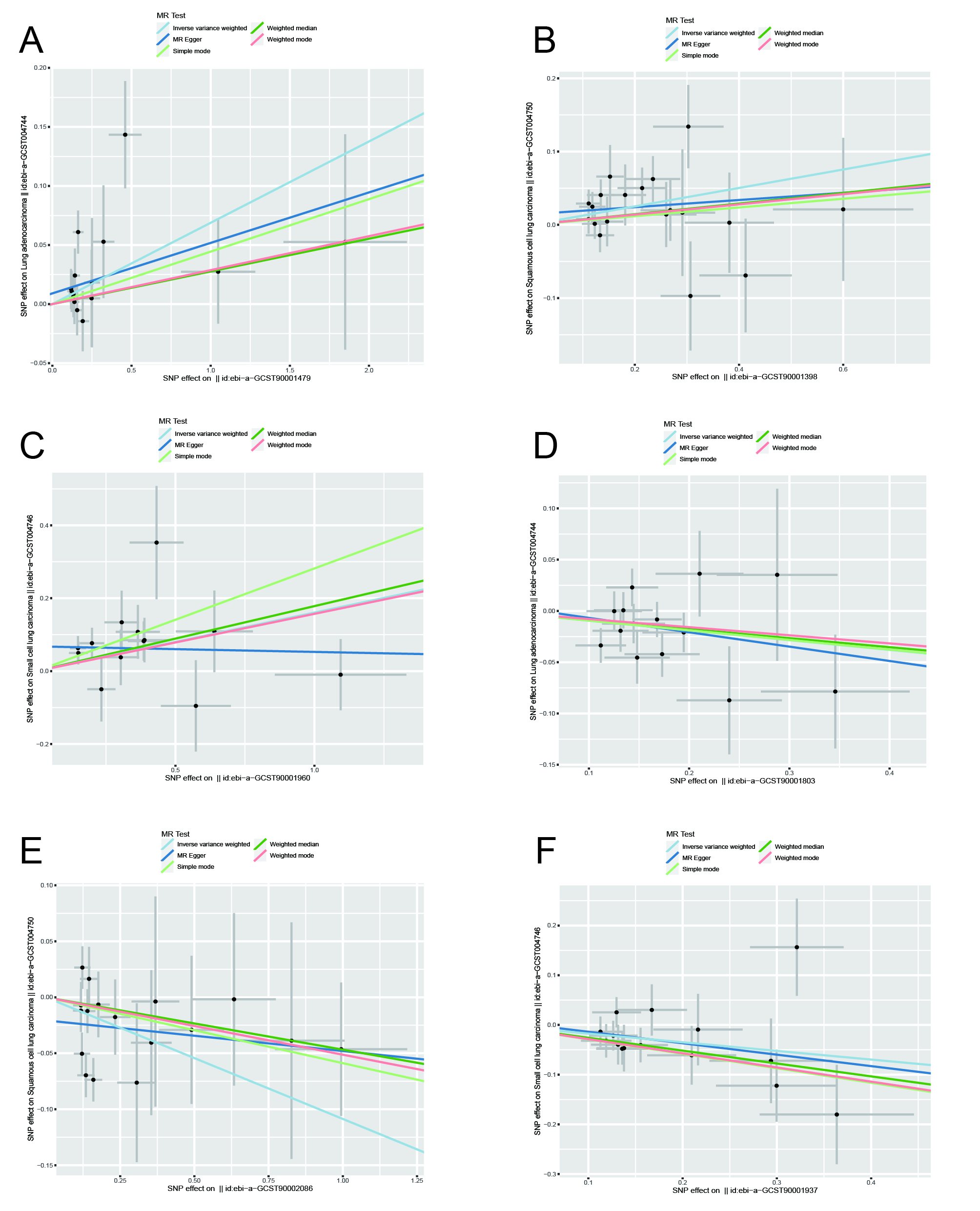

Supplement: Supplementary Figure 1 — Scatter plots illustrating genetic associations of six distinct immunophenotypes with lung cancer risk across different subtypes: (A) CD4 Treg %T cells in LUAD, (B) Unsw mem AC in LUSC, (C) CD25 on CD4+ T cells in SCLC, (D) CD27 on IgD- CD38br cells in LUAD, (E) SSC-A on HLA DR+ CD8br cells in LUSC, (F) CD25 on resting Treg cells in SCLC. [file Image_1.tif]

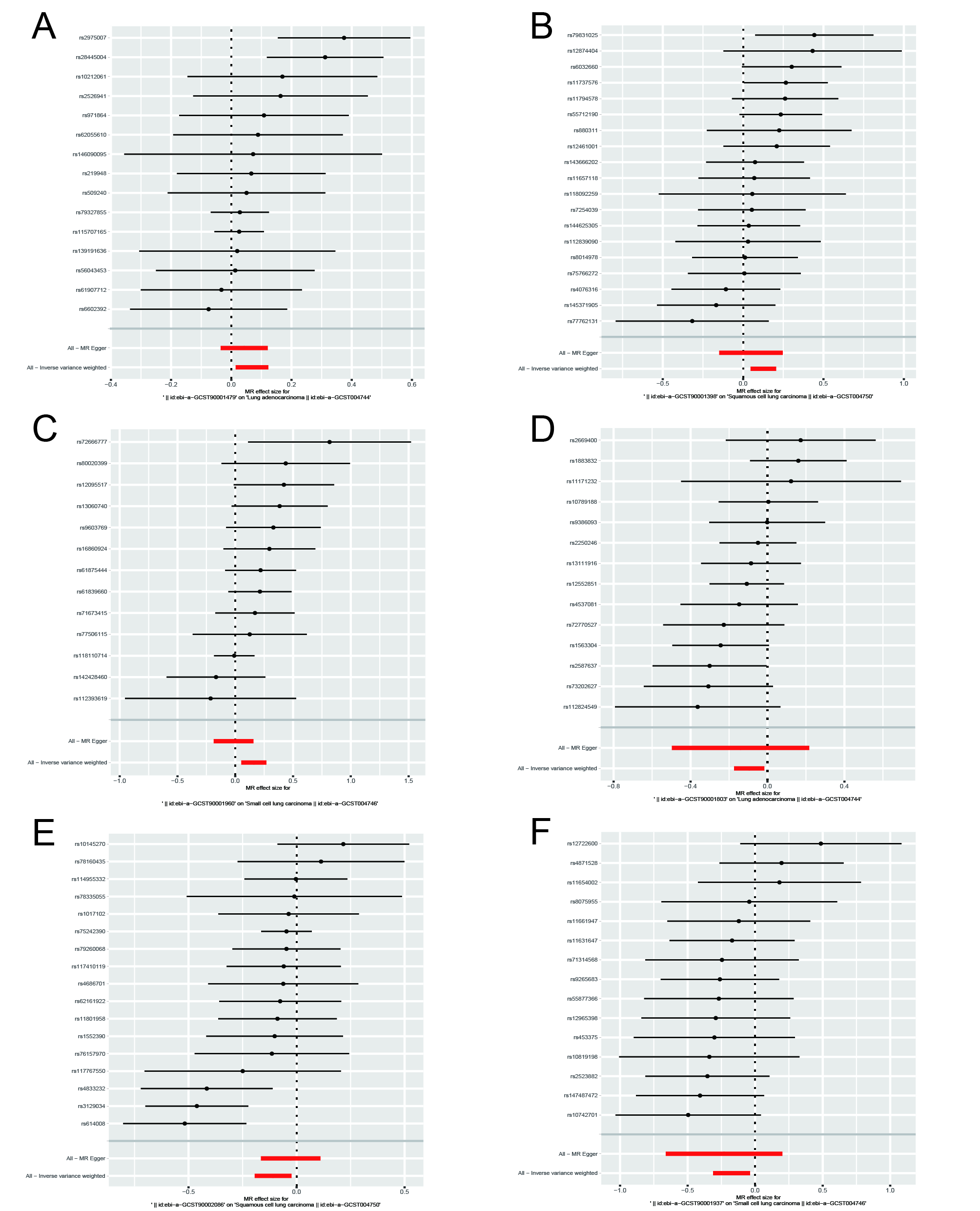

Supplement: Supplementary Figure 2 — Forest plots for six immunophenotypes in lung cancer. (A) CD4 Treg %T cells in LUAD, (B) Unsw mem AC in LUSC, (C) CD25 on CD4+ T cells in SCLC, (D) CD27 on IgD- CD38br cells in LUAD, (E) SSC-A on HLA DR+ CD8br cells in LUSC, (F) CD25 on resting Treg cells in SCLC. [file Image_2.tif]

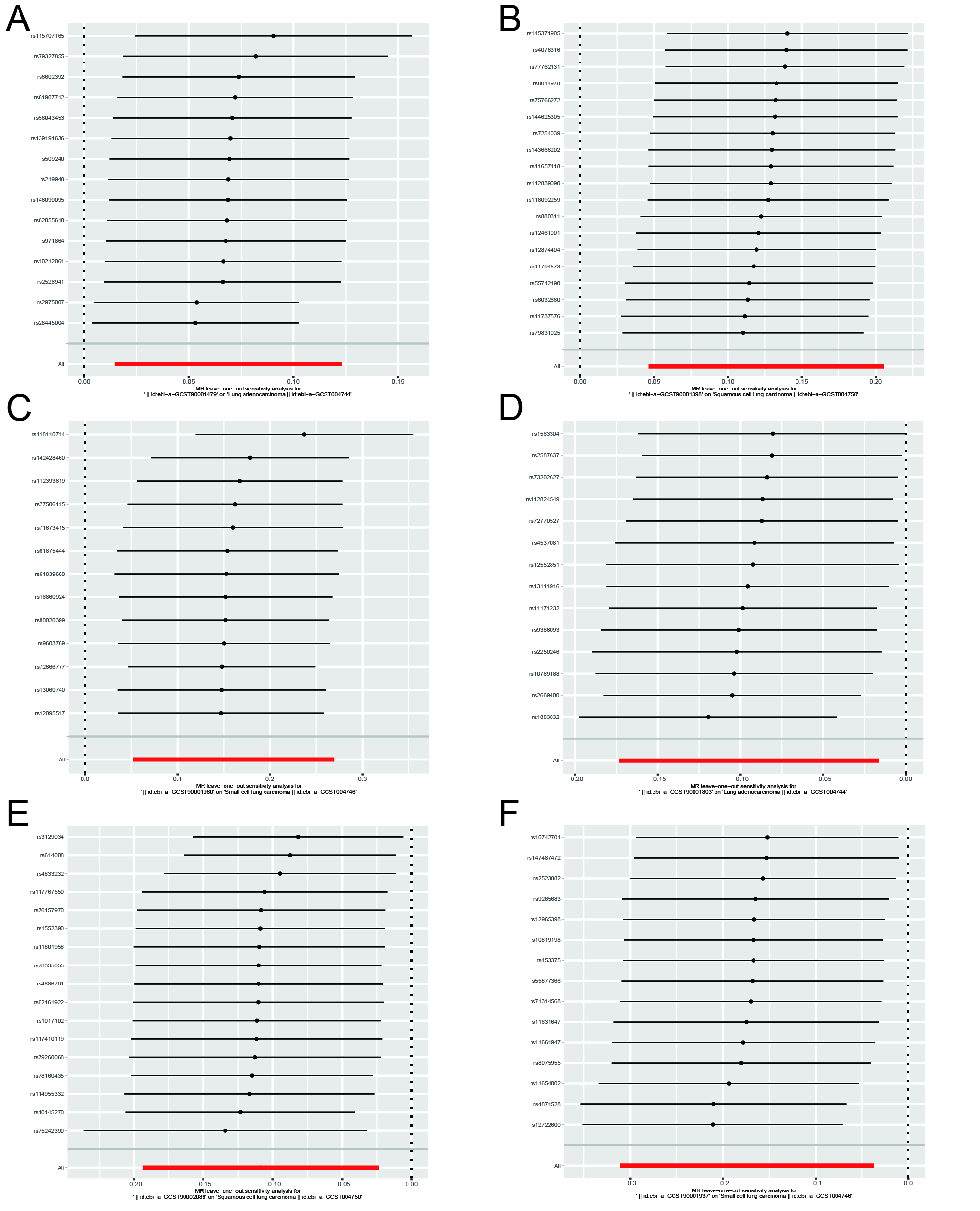

Supplement: Supplementary Figure 3 — Leave-one-out plots for six immunophenotypes in lung cancer. (A) CD4 Treg %T cells in LUAD, (B) Unsw mem AC in LUSC, (C) CD25 on CD4+ T cells in SCLC, (D) CD27 on IgD- CD38br cells in LUAD, (E) SSC-A on HLA DR+ CD8br cells in LUSC, (F) CD25 on resting Treg cells in SCLC. [file Image_3.tif]

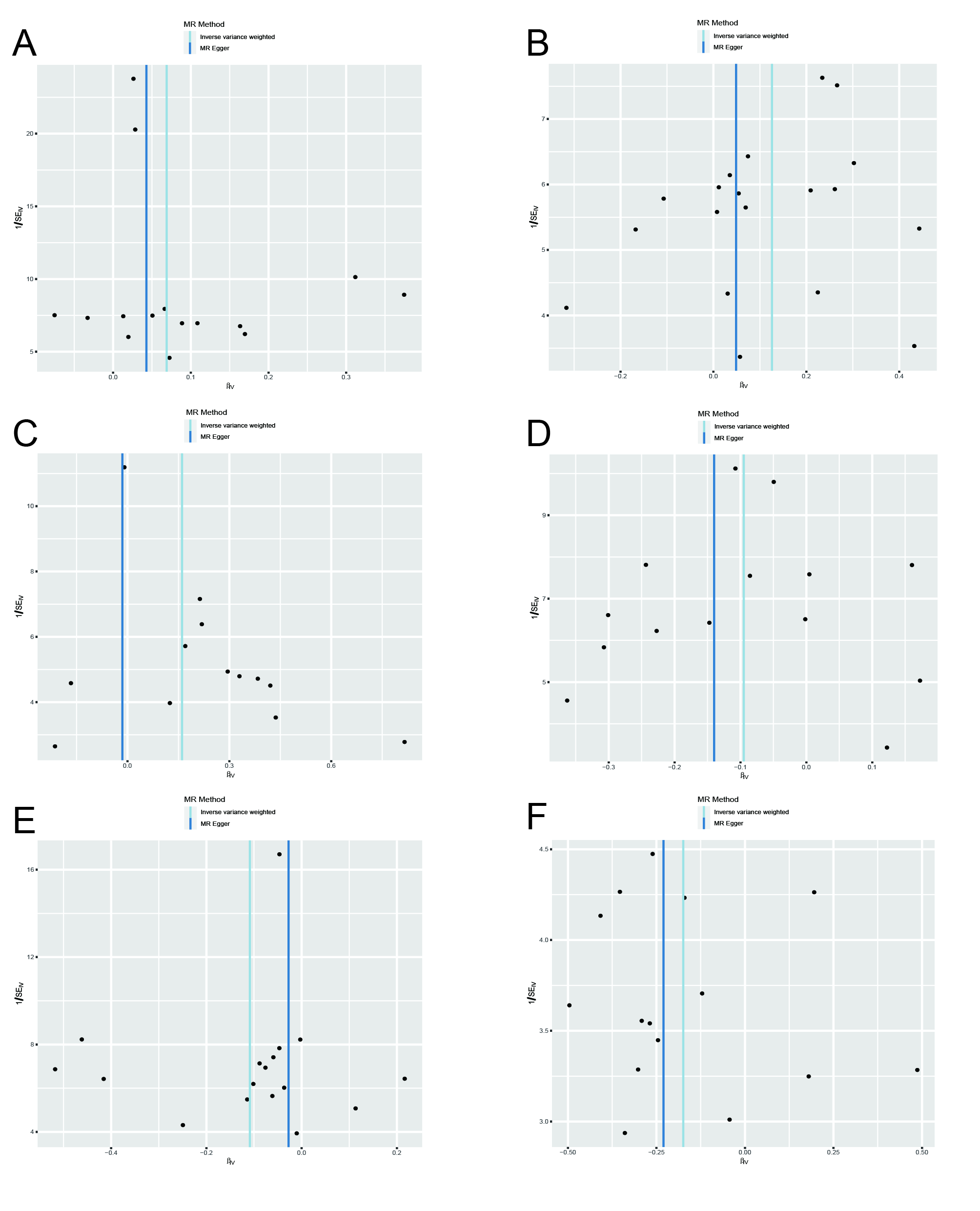

Supplement: Supplementary Figure 4 — Funnel plots for six immunophenotypes in lung cancer. (A) CD4 Treg %T cells in LUAD, (B) Unsw mem AC in LUSC, (C) CD25 on CD4+ T cells in SCLC, (D) CD27 on IgD- CD38br cells in LUAD, (E) SSC-A on HLA DR+ CD8br cells in LUSC, (F) CD25 on resting Treg cells in SCLC. [file Image_4.tif]

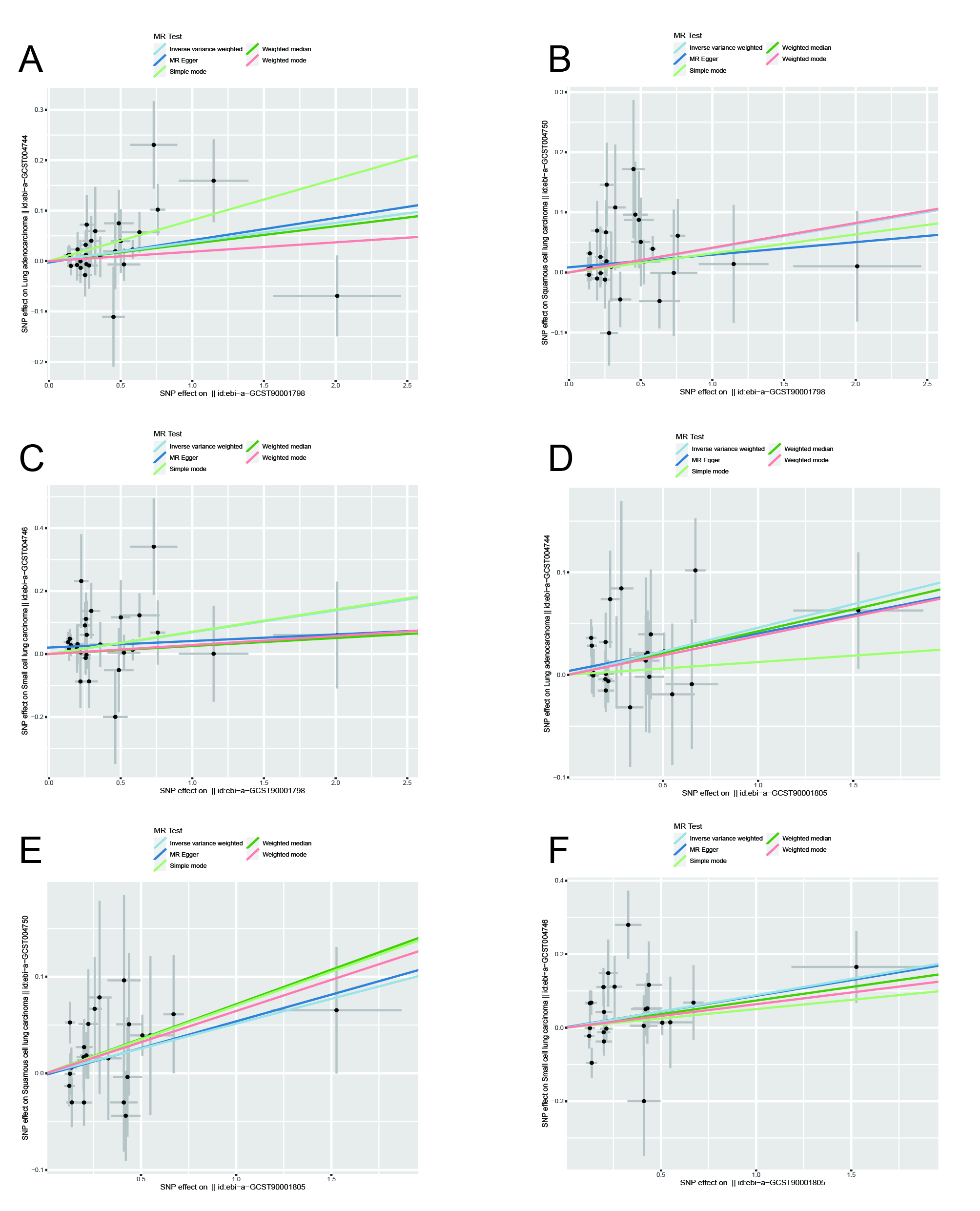

Supplement: Supplementary Figure 5 — Scatter plots depicting the genetic correlations between two immune markers and the risk of lung cancer among different subtypes. (A) CD27 on CD24+ CD27+ cells in LUAD, (B) CD27 on CD24+ CD27+ cells in LUSC, (C) CD27 on CD24+ CD27+ cells in SCLC, (D) CD27 on memory B cells in LUAD, (E) CD27 on memory B cells in LUSC, and (F) CD27 on memory B cells in SCLC. [file Image_5.tif]

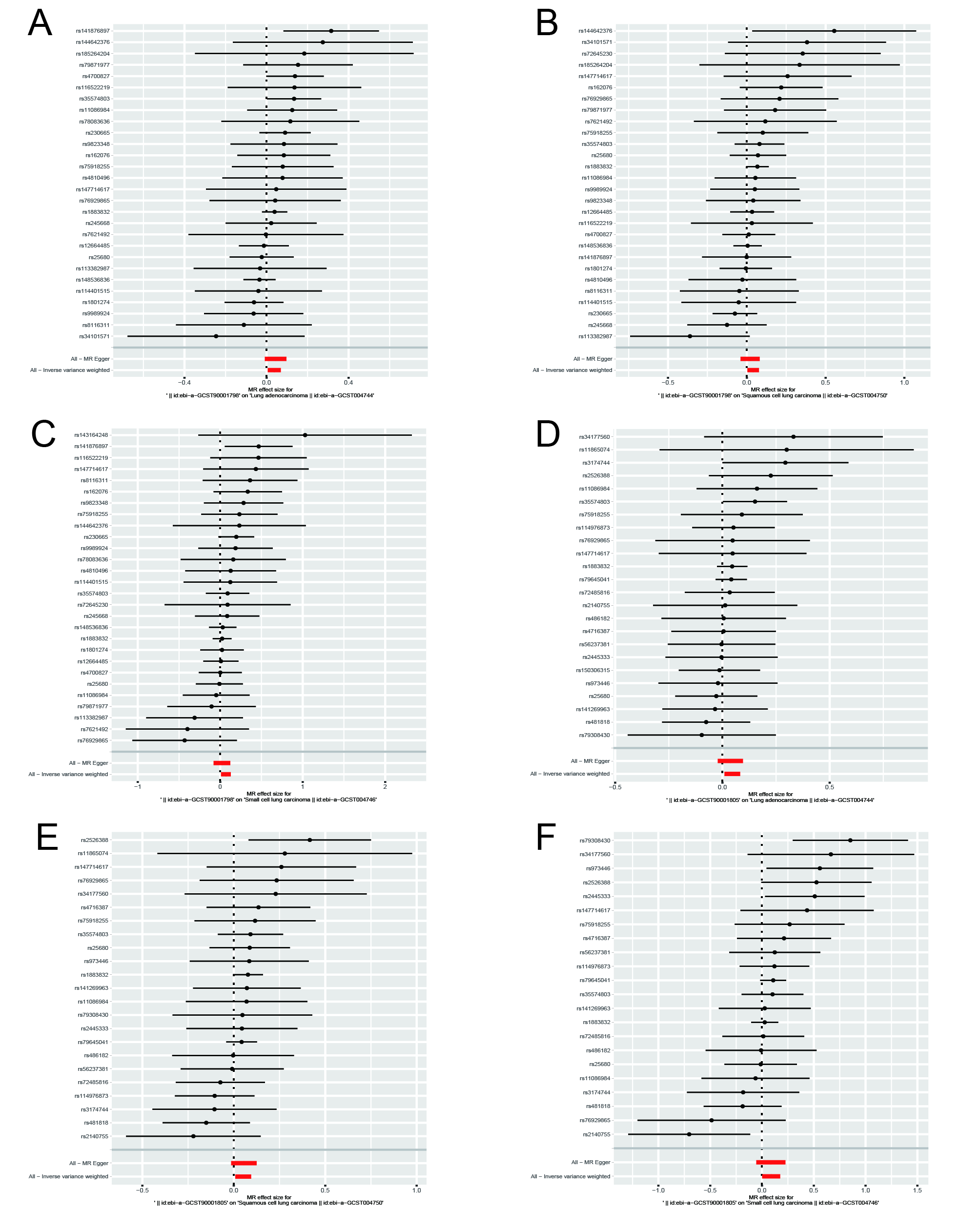

Supplement: Supplementary Figure 6 — Forest plots for assessing the association of two immune phenotypes with lung cancer risk. (A) CD27 on CD24+ CD27+ cells in LUAD, (B) CD27 on CD24+ CD27+ cells in LUSC, (C) CD27 on CD24+ CD27+ cells in SCLC, (D) CD27 on memory B cells in LUAD, (E) CD27 on memory B cells in LUSC, and (F) CD27 on memory B cells in SCLC. [file Image_6.tif]

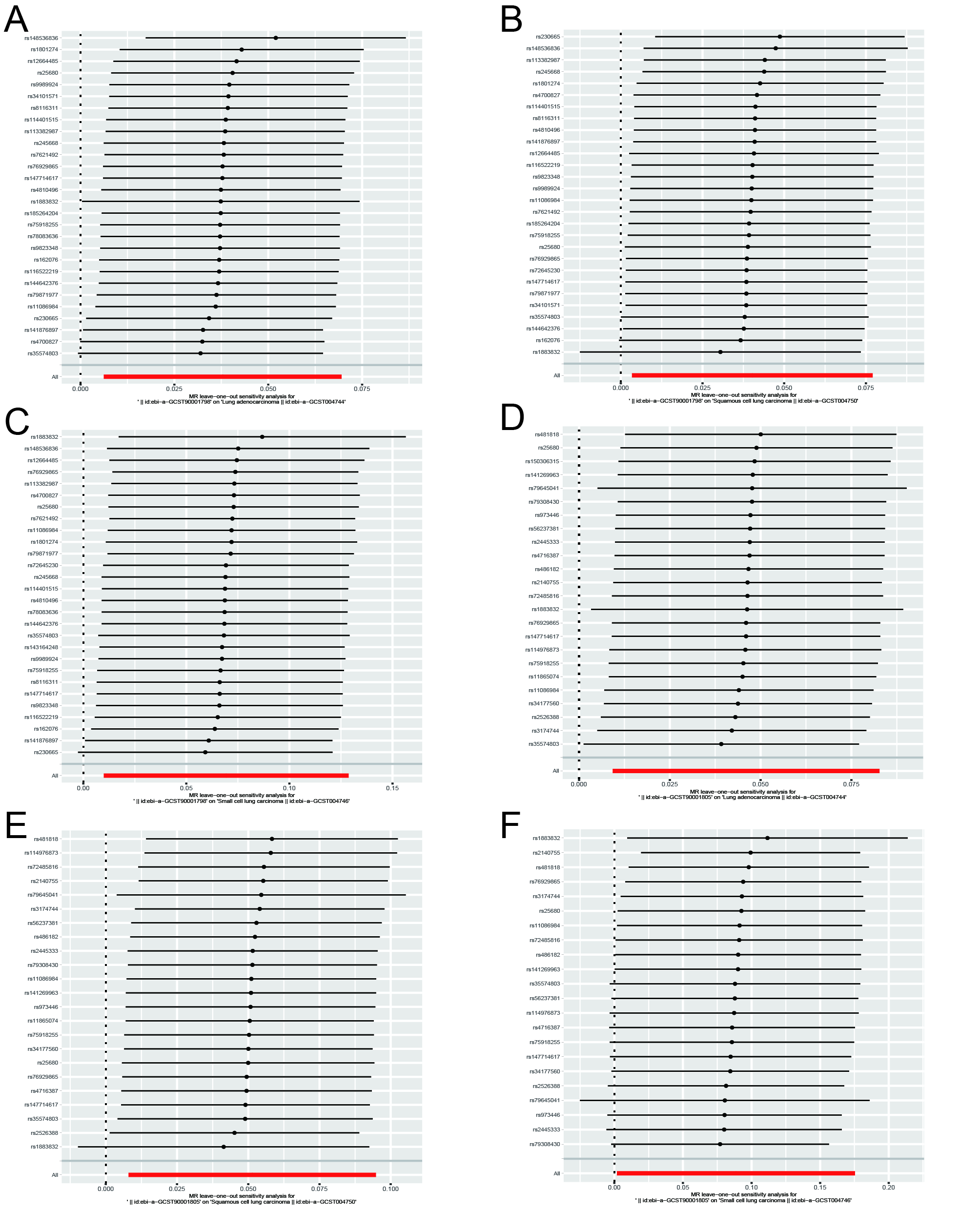

Supplement: Supplementary Figure 7 — Leave-one-out sensitivity plots for two immunophenotypes across lung cancer subtypes. (A) CD27 on CD24+ CD27+ cells in LUAD, (B) CD27 on CD24+ CD27+ cells in LUSC, (C) CD27 on CD24+ CD27+ cells in SCLC, (D) CD27 on memory B cells in LUAD, (E) CD27 on memory B cells in LUSC, and (F) CD27 on memory B cells in SCLC. [file Image_7.tif]

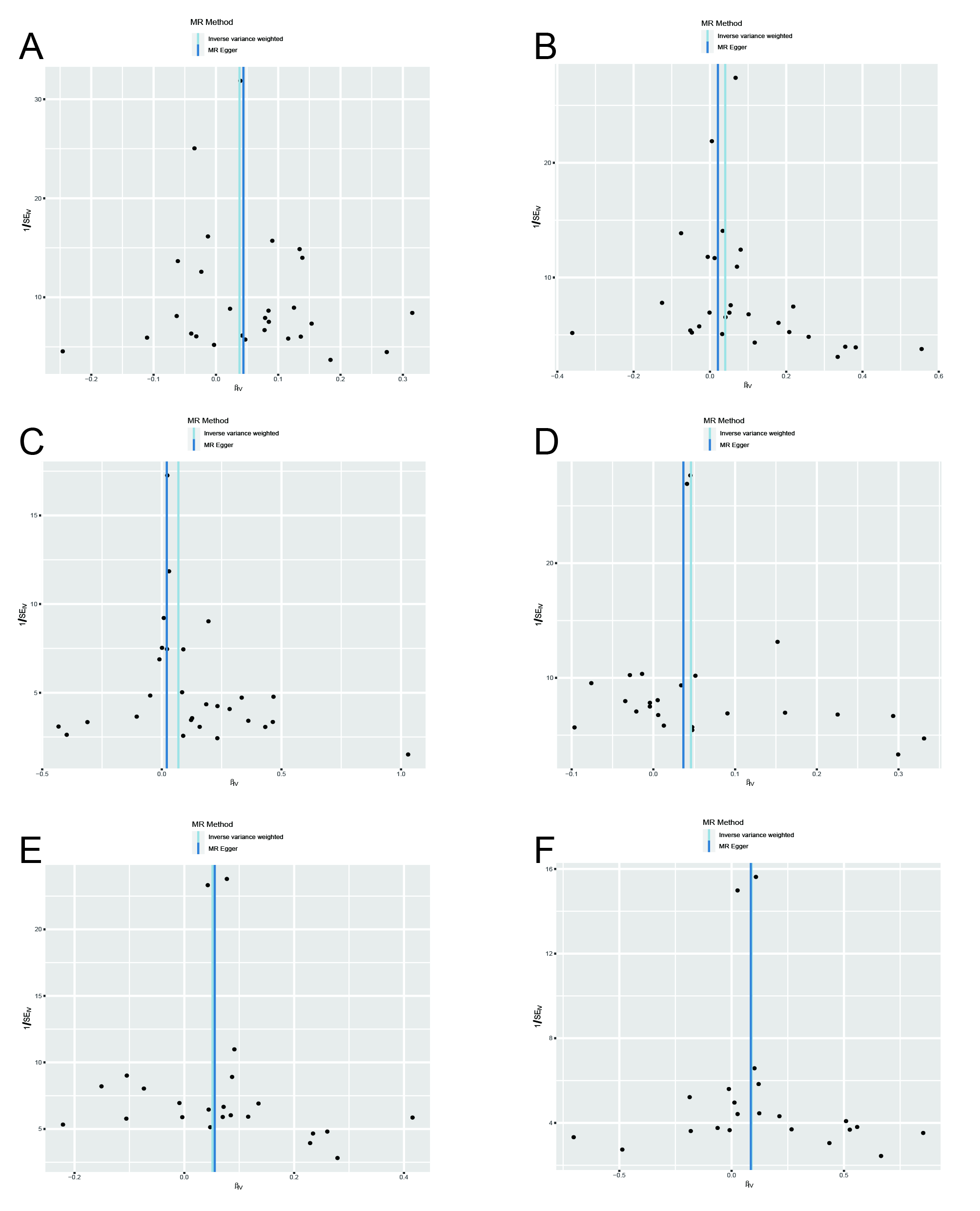

Supplement: Supplementary Figure 8 — Funnel plots for two immunophenotypes of lung cancer. (A) CD27 on CD24+ CD27+ cells in LUAD, (B) CD27 on CD24+ CD27+ cells in LUSC, (C) CD27 on CD24+ CD27+ cells in SCLC, (D) CD27 on memory B cells in LUAD, (E) CD27 on memory B cells in LUSC, and (F) CD27 on memory B cells in SCLC. [file Image_8.tif]

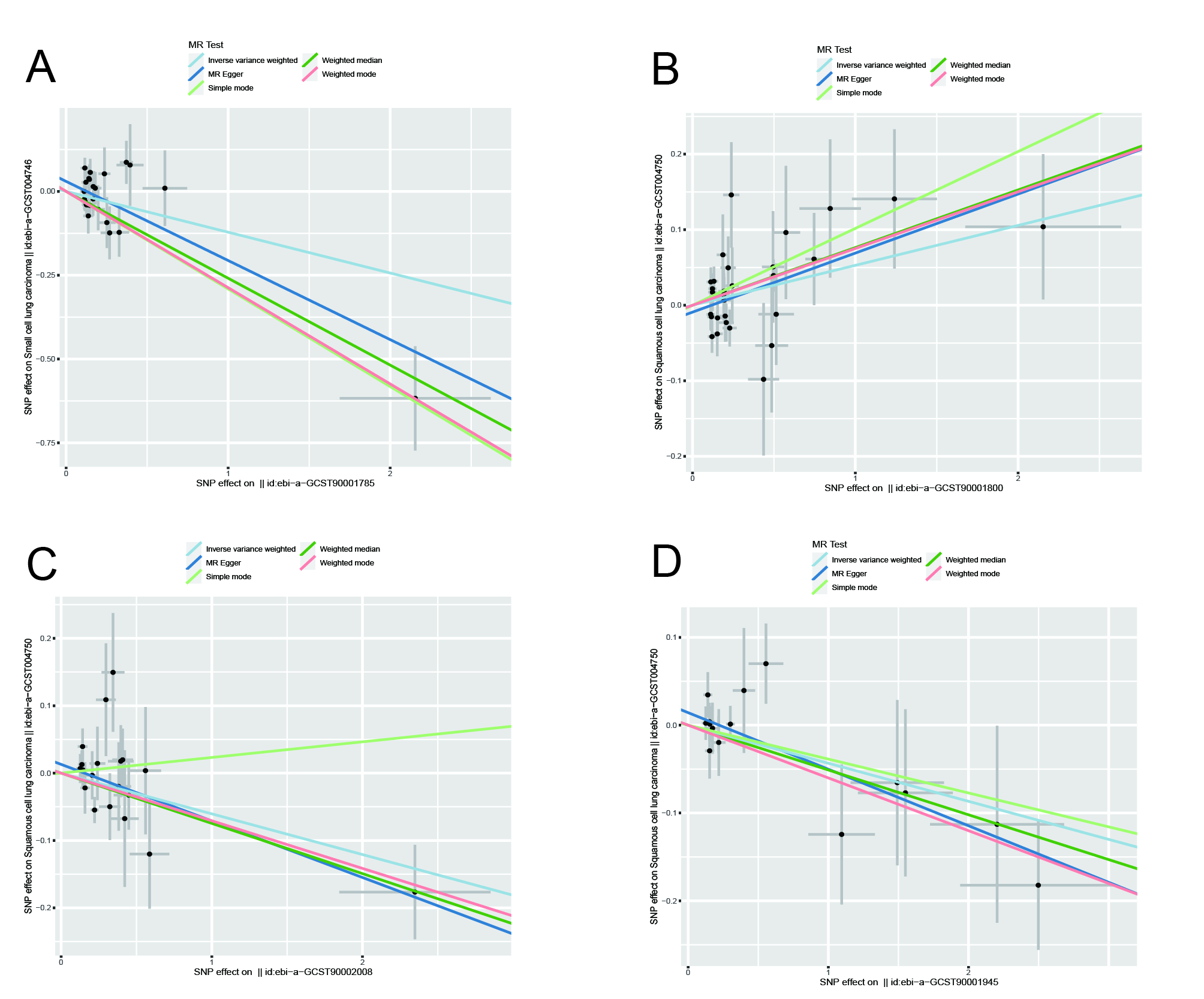

Supplement: Supplementary Figure 9 — Scatter plots depicting the genetic correlations between four immune markers and the risk of lung cancer among different subtypes. (A) CD25 on IgD- CD24- cells in SCLC, (B) CD27 on IgD+ CD24+ cells in LUSC, (C) CCR2 on monocyte cells in LUSC, and (D) CD123 on CD62L+ plasmacytoid dendritic cells in LUSC. [file Image_9.tif]

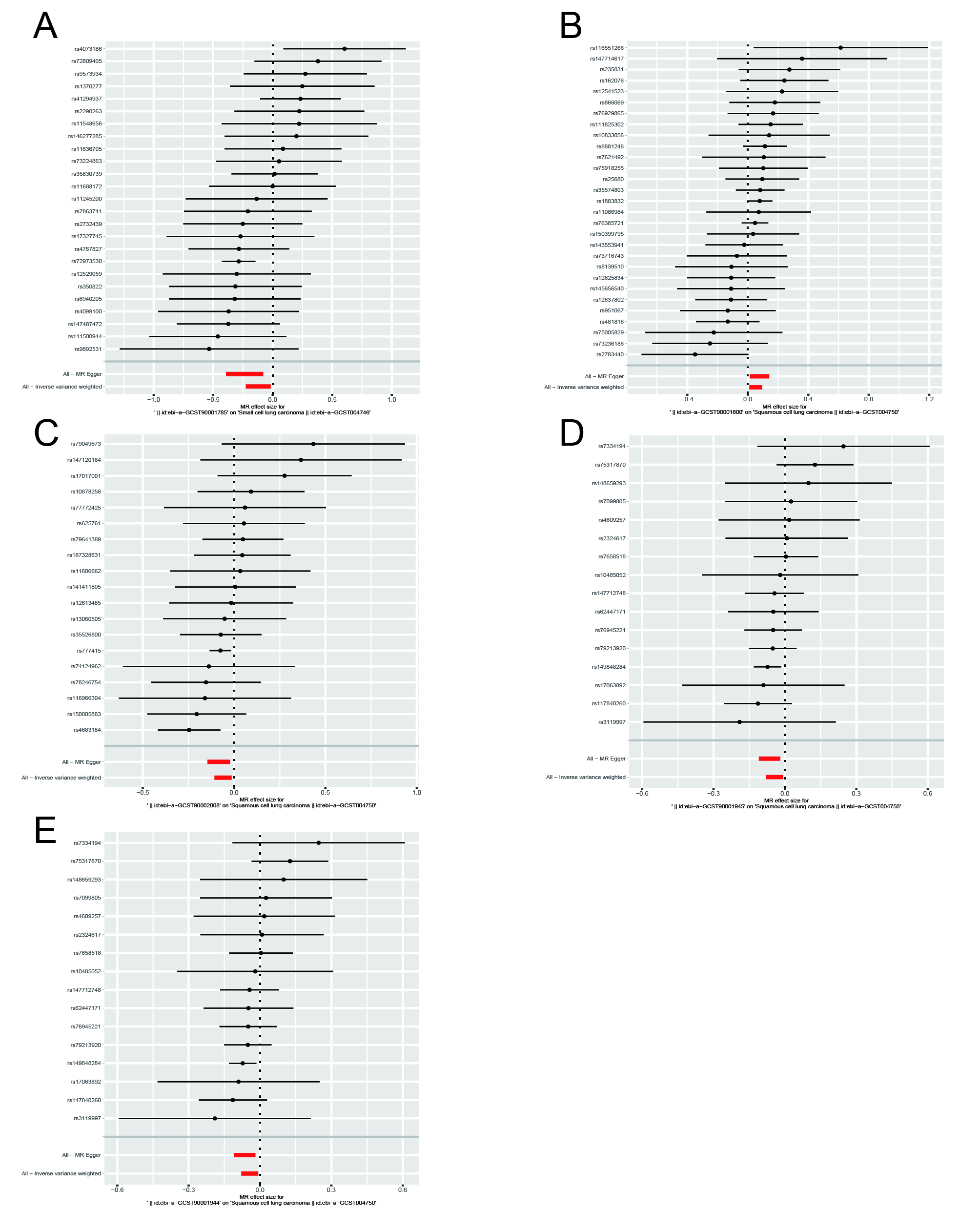

Supplement: Supplementary Figure 10 — Forest plots for assessing the association of five immune phenotypes with lung cancer risk. (A) CD25 on IgD- CD24- cells in SCLC, (B) CD27 on IgD+ CD24+ cells in LUSC, (C) CCR2 on monocyte cells in LUSC, (D) CD123 on CD62L+ plasmacytoid dendritic cells in LUSC, and (E) CD123 on plasmacytoid dendritic cells in LUSC. [file Image_10.tif]

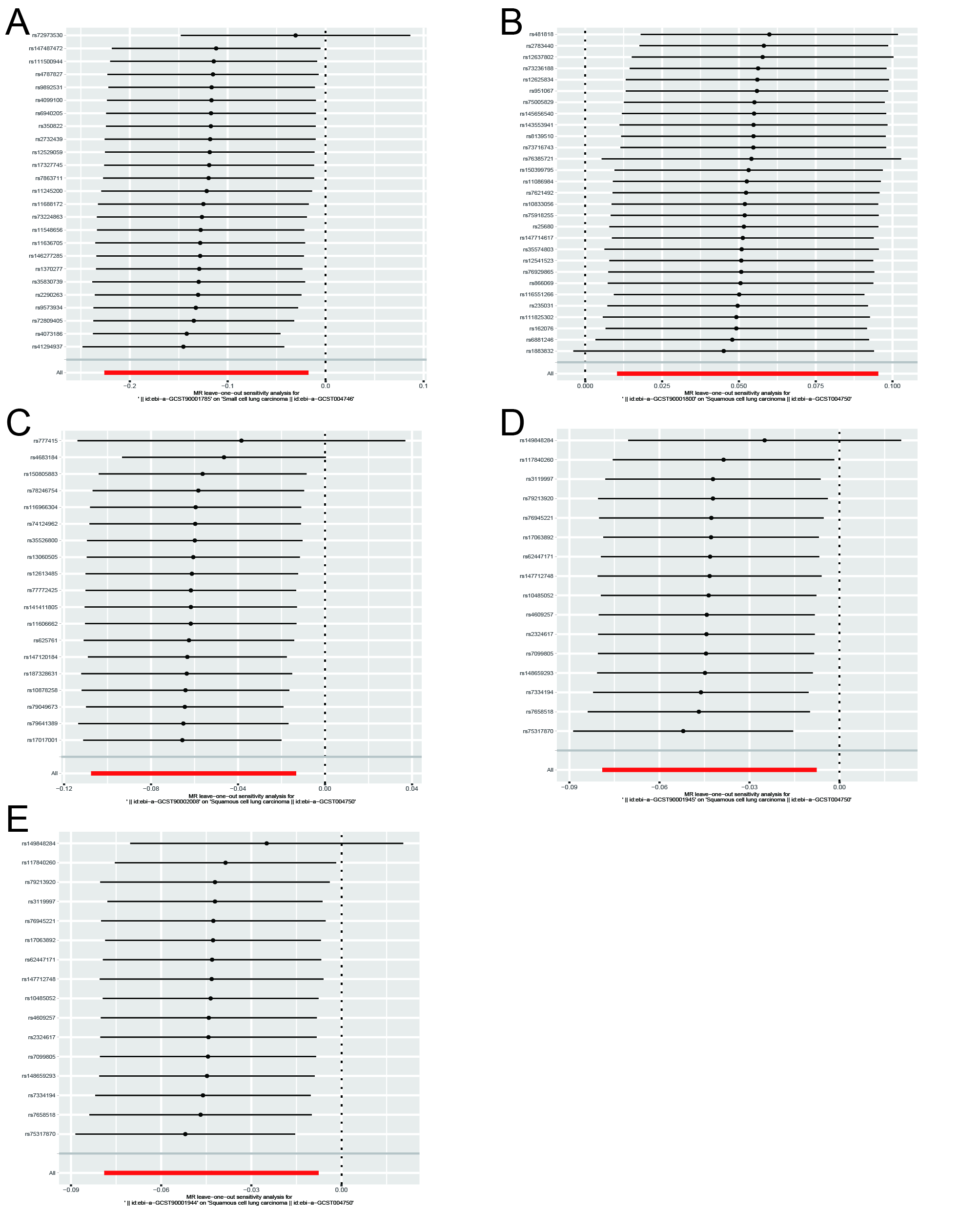

Supplement: Supplementary Figure 11 — Leave-one-out sensitivity plots for five immunophenotypes across lung cancer subtypes. (A) CD25 on IgD- CD24- cells in SCLC, (B) CD27 on IgD+ CD24+ cells in LUSC, (C) CCR2 on monocyte cells in LUSC, (D) CD123 on CD62L+ plasmacytoid dendritic cells in LUSC, and (E) CD123 on plasmacytoid dendritic cells in LUSC. [file Image_11.tif]

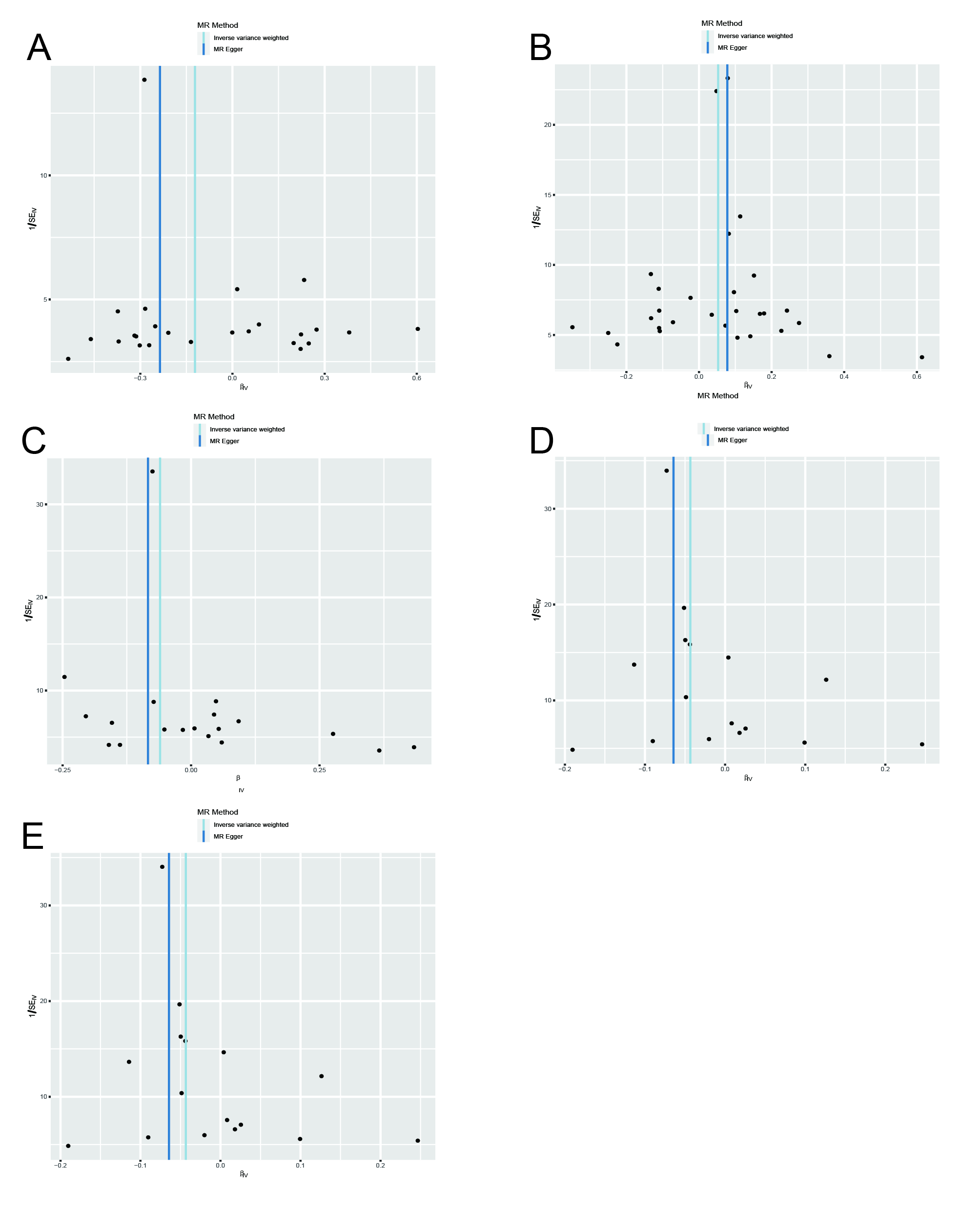

Supplement: Supplementary Figure 12 — Funnel plots for five immunophenotypes of lung cancer. (A) CD25 on IgD- CD24- cells in SCLC, (B) CD27 on IgD+ CD24+ cells in LUSC, (C) CCR2 on monocyte cells in LUSC, (D) CD123 on CD62L+ plasmacytoid dendritic cells in LUSC, and (E) CD123 on plasmacytoid dendritic cells in LUSC. [file Image_12.tif]
